# Maternal obesity and diabetes may cause DNA methylation alteration in the spermatozoa of offspring in mice

**DOI:** 10.1186/1477-7827-12-29

**Published:** 2014-04-11

**Authors:** Zhao-Jia Ge, Qiu-Xia Liang, Yi Hou, Zhi-Ming Han, Heide Schatten, Qing-Yuan Sun, Cui-Lian Zhang

**Affiliations:** 1 Reproductive Medicine Center, Henan Provincial People’s Hospital, Zhengzhou 450003, Henan Province, P.R. China; 2 State Key Laboratory of Reproductive Biology, Institute of Zoology, Chinese Academy of Sciences, Beijing 100101, P.R. China; 3 Reproductive Medicine Center, People’s Hospital of Zhengzhou University, Zhengzhou 450003, Henan province, P.R. China; 4 Department of Veterinary Pathobiology, University of Missouri, 65211 Columbia, MO, USA

**Keywords:** Spermotozoa, Offspring, Methylation, Maternal diabetes/obesity

## Abstract

**Background:**

The adverse effects on offspring of diabetic and/or obese mothers can be passed to the next generation. However, the mechanisms behind this are still unclear. Epigenetics may play a key role during this process.

**Methods:**

To confirm the hypothesis, we investigated the DNA methylation of several imprinted genes in spermatozoa of offspring from diabetic and/or obese mothers utilizing streptozotocin (STZ)- and high-fat-diet (HFD)-induced mouse models.

**Results:**

We found that the DNA methylation of *Peg3* was significantly increased in spermatozoa of offspring of obese mothers compared to that in spermatozoa of offspring of normal mothers. The DNA methylation of *H19* was significantly higher in spermatozoa of offspring of diabetic mothers than that in spermatozoa of offspring of non-diabetic mothers.

**Conclusions:**

These results indicate that pre-gestational diabetes and/or obesity can alter DNA methylation in offspring spermatozoa.

## Background

There is evidence that exposure to an adverse fetal and/or early postnatal environment may enhance susceptibility to a number of chronic diseases in the future life of offspring. Pre-existing maternal diabetes and obesity are both complications which influence the development of offspring during fetal life and postnatal development. Offspring of women with pre-gestational diabetes have a high risk of developing obesity, impaired glucose tolerance, and type 2 diabetes in adulthood [1]. Epidemiological and clinical studies have shown that maternal type 1 diabetes during pregnancy is an important risk factor for the development of obesity and diabetes in offspring [2,3]. In animal models, a fetus exposed to poorly controlled female diabetes mellitus has a higher incidence of abortion, metabolic diseases, malformation, and stillbirth [4,5]. Furthermore, offspring from females with pregestational diabetes are susceptible to onset of obesity, glucose intolerance, and type 2 diabetes [1,6]. Such metabolic consequences persist through the F1 and F2 generations [7,8].

It is widely recognized that obesity is becoming a big problem for the world-wide. Offspring of women with pre-pregnant obesity and overweight are prone to onset of obesity, type 2 diabetes, hypertension, and cardiovascular diseases [9]. A retrospective study shows that children of obese mothers are twice as likely to be obese at 24 months of age compared to non-obese mothers [10]. Lawlor and colleagues showed that offspring of overweight and obese mothers had more adipose tissue than those born to normal-weight mothers at 18 years of age [10,11]. In high fat diet-induced obese rats, the offspring of obese dams exhibit increased adiposity and insulin resistance until postnatal day 130 compared to those of lean dams [12]. Bayol et al. showed that 10-week-old rats born to mothers fed a high-fat diet exhibited an increased body weight and fat mass when compared to control offspring [13]. Obesity, insulin resistance, glucose intolerance and metabolic syndrome observed in offspring born by obese mothers are still apparent in the second generation of offspring [14].

The intergenerational transmission of adverse health effects from mothers with pre-existing diabetes and/or obesity cannot be completely explained by genetics [15]. Epigenetics may perfectly illustrate how the effect is inherited by the next generations. It has been reported that maternal and/or paternal lineages imprinted genes may play a key role in defects perpetuated to the next generations [16,17]. In our study, we have previously shown that the methylation patterns of several imprinted genes in oocytes of offspring from mothers with pregestational diabetes [18] or obesity [19] were not altered. However, the overall methylome patterns in male pup’s sperm of pre-diabetic fathers were altered [20]. Whether the DNA methylation status in male offspring spermatozoa of pre-diabetic/-obese mothers is affected is still unknown. Therefore, we investigated the DNA methylation patterns in DMRs (differential methylation regions) of paternally imprinted genes *H19, Gtl2*, *Rasgrf1*, and maternally imprinted genes *Peg3*, *Snrpn* in spermatozoa of offspring born to obese mothers. In this study we further investigated DNA methylation patterns of *H19, Gtl2* and *Peg3* in spermatozoa from offspring of diabetic mothers.

## Materials

CD-1 mice were provided by Beijing Vital River Experimental Animals Centre and fed in a temperature controlled room with a light cycle of 12 L:12D (light:dark). All procedures described were reviewed and approved by the ethical committee of the Institute of Zoology, Chinese Academy of Sciences.

### Offspring born by maternal diabetic and obese mice

CD-1 mice 6-7-weeks old whose weights were 26.5-27.5 g were divided into two groups, randomly. One group received a single intraperitoneal injection of streptozotocin (STZ), which can impair islets and reduce the synthesis of insulin, at a dose of 230 mg/kg and the other group received buffer. The blood glucose level was checked utilizing a glucometer, Blood Testing Equipment, Accu-CHEK Active (Roche Diagnostic, Germany) by cutting the tip of the tail on day 4 after injection of STZ. If the glucose concentration was higher than 17.0 mmol/l, the mice were selected. The blood glucose level of mice injected with buffer was also checked and the blood glucose level was lower than 7.0 mmol/l. The diabetic and nondiabetic mice were mated with normal male mice at 15 days of injection with STZ/buffer. Twenty one days later, offspring were born. At the age of 7–8 weeks, nine male pups of 3–4 litters of diabetic and nondiabetic group were randomly selected and analyzed.

**Figure 1:**
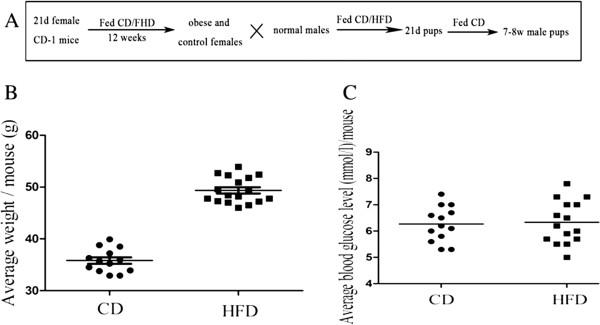
**Average weight and blood glucose level in high-fat-diet (HFD)-induced mouse models. (A)** At age of 21d (day), the female mice were randomly divided into two groups and fed with CD (control diet) and HFD (high-fat-diet), respectively. Twelve weeks later, mice fed with CD/HFD were mated with normal male mice, respectively. The pups of them were fed with diets as pre-pregnancy until weaning. Weaned male pups were fed with CD. At age of 7–8 weeks, spermatozoa of male pups were collected. After 12 weeks of treatment, the average weight of the HFD (n = 17) group was clearly heavier than that of the CD (n = 13) group, P < 0.001 **(B)**, but the average blood glucose level of CD (n = 13) and HFD (n = 15) was similar between the two groups **(C)**, P = 0.8209.

The weaned CD-1 mice were divided randomly into two groups. One group was fed with high-fat-diet (HFD, D12492, fat: 60% kcal, carbohydrate: 20% kcal, protein: 20% kcal, Research Diets, America) and the other was fed with control diet (CD, fat: 9.3% kcal, carbohydrate; 70% kcal, protein: 20% kcal) for 12 weeks. Then the mice fed with HFD or CD was mated with male mice fed normal diets to produce offspring, respectively. During gestational and lactational periods, the females were fed with HFD and/or CD as pre-pregnancy. After weaning, all offspring were fed CD. At the age of 7–8 weeks (Figure 1A), a total of 9–10 male pups of 3–4 litters were randomly selected and analyzed for HFD and CD.

### Offspring sperm collection

Spermatozoa of offspring were collected according to previously described method [21]. Briefly, the cauda epididymis was separated and punctured with a sterile needle in HTF (Human Tube Fluid) for 30 min at 37°C. The motile spermatozoa that remained in the supernatant were carefully transferred to a new Eppendorf tube (EP) and this procedure was repeated 3 times. Then the supernant was centrifuged at 13,400 rpm for 10 min to pellet the sperm.

### DNA bisulfite modification and PCR amplification

Spermatozoa were modified with EZ DNA Methylation-Direct™ Kit (ZYMO RESEARCH, USA) according to the manufacturer’s direction. Modified DNA was used as template for nested-PCR amplification. Briefly, nested PCR was carried out using 0.5 μl solution with modified DNA in the first round PCR and 2 μl product of first round PCR was added into the second reaction system as template. The relative primers are shown in Table 1.

**Table 1:** Oligonucleotides utilized for PCR

**Genes**	**Primer name**	**Primer sequence**
***H19***	Forward1	5′-GAGTATTTAGGAGGTATAAGAATT-3′
	Reverse1	5′-ATCAAAAACTAACATAAACCCCT-3′
	Forward2	5′-GTAAGGAGATTATGTTTATTTTTGG-3′
	Reverse2	5′-CCTCATTAATCCCATAACTAT-3′
***Peg3***	Forward1	5′-TGATAATAGTAGTTTGATTGGTAGGG-3′
	Reverse1	5′-TAATTCACACCTAAAACCCTAAAACC-3′
	Forward2	5′-TTTTGTAGAGGATTTTGATAAGGAGG-3′
	Reverse2	5′-AAATACCACTTTAAATCCCTATCACC-3′
***Snrpn***	Forward1	5′-TATGTAATATGATATAGTTTAGAAATTAG-3′
	Reverse1	5′-AATAAACCCAAATCTAAAATATTTTAATC-3′
	Forward2	5′-AATTTGTGTGATGTTTGTAATTATTTGG-3′
	Reverse2	5′-ATAAAATACACTTTCACTACTAAAATCC-3′
***Gtl2***	Forward1	5′-GGGAATAGGATGTATTATGGAGTAATG-3′
	Reverse1	5′-ATATACCACATAACTATACCA-3′
	Forward2	5′-GGTTAAGTGGTTTGTAGTAT-3′
	Reverse2	5′-ATATACCACATAACTATACCA-3′
***Rasgrf1***	Forward1	5′-TAATTTTAGGTGTAGAATATGGGGTTG-3′
	Reverse1	5′-TAAAAAAACAAAAACAACAATAACAACTAAAACAAAAACAA-3′
	Forward2	5′-TAGAGAGTTTATAAAGTTAG-3′
	Reverse2	5′-ACTAAAACAAAAACAACA-3′
***sH19***	Forward1	5′-AAATTTTAATTTTGGTTGTTTTTGG-3′
	Reverse1	5′-AATCAATTAAAAAAATAATAAAACCC-3′
	Forward2	5′-TGGTTGTTTTTGGAATATAATGTT-3′
	Reverse2	5′-AAAAACAAAACACCTATACCCTTC-3′

### Combined bisulfite restriction analysis (COBRA) and bisulfite sequencing (BS)

When DNA was treated by bisulfite, the unmethylated CG was translated to TG. So we can use restriction endogenous enzymes and sequencing to analyze the methylation patterns at CpG (cytosine-phosphate-guanine) sites [18]. The product of nested-PCR was digested by one or two restriction endogenous enzymes of which there were CpG loci included in the recognition sites. The recognition sites of enzymes used in the assay were *Taq*^*α*^I (T/CGA), *Rsa*I (GTAC/), and *Bst*UI (CG/CG). To further investigate the methylation patterns of relative genes, all samples were pooled together and was cloned using T vector (TAKARA). Then it was sequenced. At least ten clones per gene were sequenced.

### Statistical analysis

Body weight and blood glucose are represented as Mean ± SD and the significance between groups was evaluated by One-way ANOVA. The Chi-square test was used to evaluate whether significant difference exists in methylation density between different groups. A probability level of P < 0.05 was considered significant.

## Results

### DNA methylation in spermatozoa of offspring of obese mothers is altered

**Figure 2:**
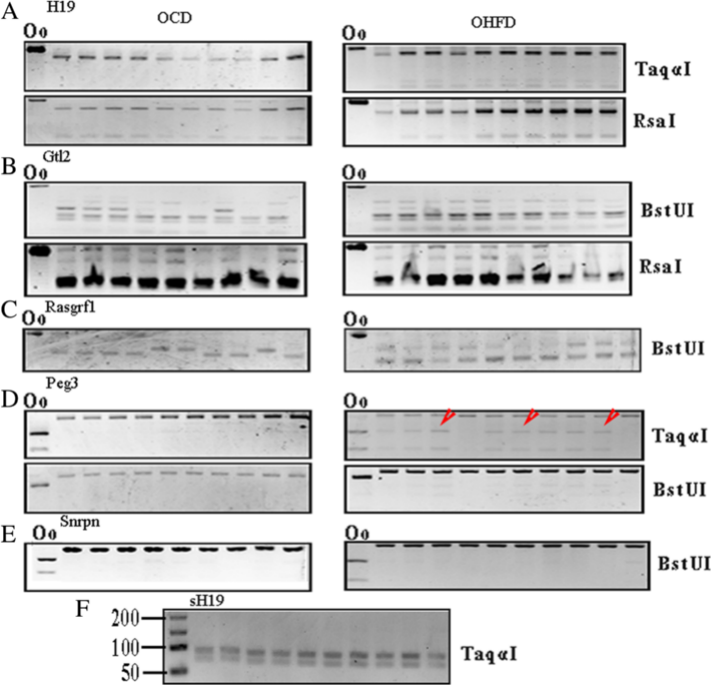
**DNA methylation status in DMRs of imprinted genes in spermatozoa of OHFD analyzed by COBRA.** DNA methylation patterns in DMRs of paternally imprinted genes *H19* (423 bp)*, Gtl2* (425 bp), *Rasgrf1* (284 bp) and maternally imprinted genes *Peg3* (444 bp) and *Snrpn* (420 bp) in spermatozoa of OHFD (n = 9-10) and OCD mice (n = 10) were evaluated by COBRA. Oocytes were utilized as a control. Enzymes used are shown in the right column; Oo, oocyte. **(A)***H19*; **(B)***Gtl2*; **(C)***Rasgrf1*; **(D)***Peg3*, red arrowheads showed the digested bands; **(E)***Snrpn*; **(F)** a shorter region located in DMR of *H19* was amplified with *Peg3* in the first-round PCR and the methylation pattern was analyzed by COBRA.

After 12 weeks fed with CD/HFD, the average weight of mice fed with HFD (49.37 ± 0.6037) was obviously heavier than those fed with CD (35.81 ± 0.6295, Figure 1B). The average blood glucose level between HFD and CD groups was similar (Figure 1C). A total of 9–10 pups of obese/non-obese mothers were analyzed in each group. The pups were randomly selected from 3–4 litters. For paternally imprinted genes, results of COBRA showed that all of them were digested by relative enzymes (Figure 2A-C). Compared to oocytes, the bands of spermatozoa of the maternally imprinted gene *Snrpn* was completely undigested by *BstuI* (Figure 2E). This also confirmed that our samples were not contaminated by somatic cells. However, for *Peg3*, we observed that several samples were partly digested by *Taq*^*α*^I and/or *BstuI* (Figure 2D, shown by red arrowheads). To further prove that these results were not attributed to contamination by somatic cells, we carried out first-round PCR with primers of *Peg3* and *sH19* (a shorter fragment in DMR of *H19*, 176 bp). Then the products of PCR were analyzed by COBRA and the results showed that products of s*H19* were completely digested (Figure 2F).

**Figure 3:**
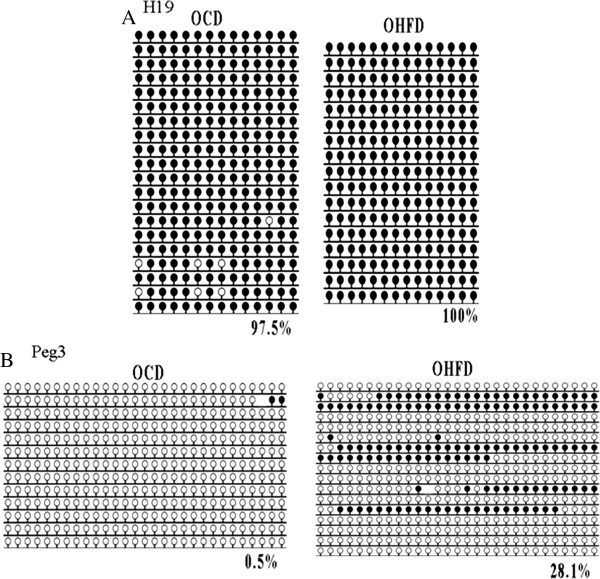
**DNA methylation in spermatozoa of OHFD analyzed by BS.** DNA methylation of paternally imprinted gene *H19* and maternally imprinted gene *Peg3* was further analyzed by BS. All the samples were pooled together for each group. **(A)** represents *H19* methylation status; the number shows the methylation%; **(B)** shows the methylation patterns of *Peg3*. Black circle, methylated; white circle, unmethylated; blank loci, CpG lost.

Next, we used BS to further investigate the methylation patterns of paternally imprinted gene *H19* and maternally imprinted gene *Peg3*. We pooled the products of all samples together for *H19* and *Peg3*, respectively. 10–20 clones were sequenced. We evaluated the bisulfite conversion rates and it ranged from 95% to 99% (the average conversion rate was 97.74%). Sequencing results showed that DNA methylation in DMR of *H19* was not affected in sperm, either (Figure 3A). For *Peg3*, the methylation level in the OHFD group was 28.05%, which is significantly higher (P < 0.01) than that in the OCD (male offspring of non-obese mothers, 0.5%) group (Figure 3B).

### DNA methylation status in DMR of paternally imprinted gene *H19* is altered in spermatozoa of offspring from diabetic mothers (OD)

**Figure 4:**
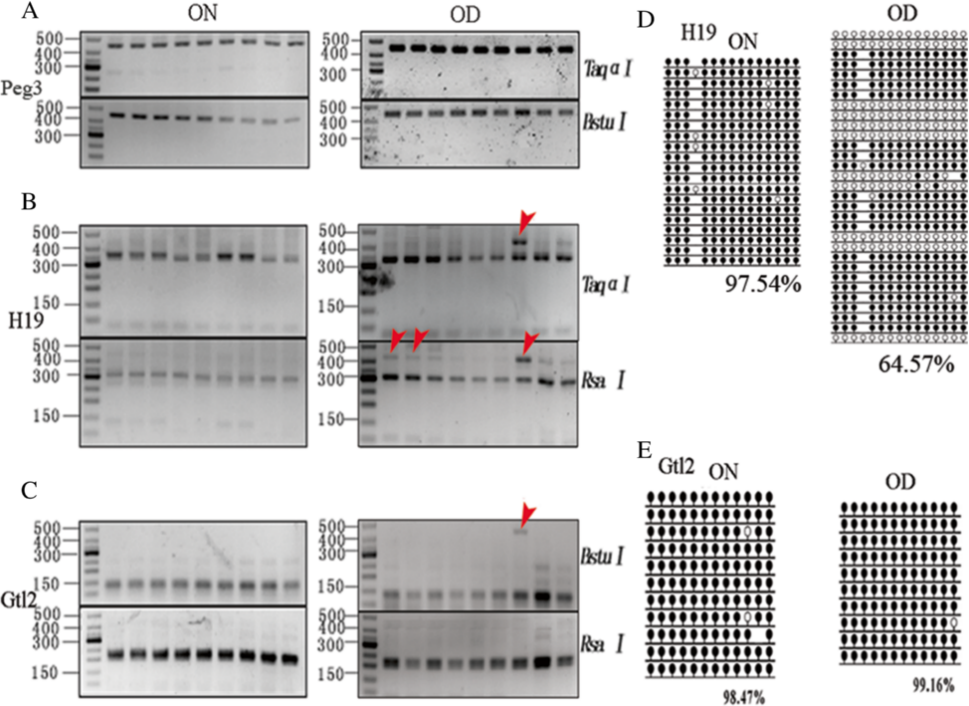
**Methylation patterns in DMRs of imprinted genes in sperm from offspring of diabetic (OD) and non-diabetic (ON) females.** OD (n = 9)and ON (n = 9) (7–8 weeks old) mice were killed and spermatozoa were collected and treated with bisulfite and then amplified by nest-PCR. **(A ~ C)** The products of PCR were digested by enzymes and analyzed by 2.5% agarose gel electrophoresis. The red arrow heads point to the samples that were only partially digested by enzymes. **(D, E)** The methylation rates in DMRs of *H19* and *Gtl2* were evaluated by sequencing. Black circle, methylated; white circle, unmethylated; blank loci, CpG lost.

We checked the blood glucose level of diabetic and non-diabetic mothers at pre-pregnancy (nondiabetic & diabetic, 6.14 ± 0.51 & 23.53 ± 3.27) and post-partum (nondiabetic & diabetic, 6.46 ± 0.61 & 25.86 ± 1.34). Dlood glucose level was similar between ON (offspring of nondiabetic mothers, 6.72 ± 0.46) and OD (offspring of diabetic mothers, 7.1 ± 0.74) at age of 7–8 weeks. Nine pups from 3–4 litters were analyzed in each group. As shown in Figure 4A, the samples of *Peg3* were all uncut by both enzymes in the two groups, suggesting that the methylation pattern of *Peg3* in spermatozoa of OD was not affected by maternal diabetes (Figure 4A). However, for *H19*, some of the samples were not all cut by both enzymes (Figure 4B) (shown by red arrows). In Figure 4C, some samples were still not completely digested by one or two enzymes.

Although some samples were not all digested by enzymes for *H19* and *Gtl2*, maybe the demethylation just took place on recognition sites. To further investigate the methylation status, we pooled all of the samples together and sequenced according to COBRA results. The sequencing results indicated that the methylation level of *H19* in spermatozoa of OD (64.57%)was significantly lower than that of ON (97.54%, P < 0.001, Figure 4D). For *Gtl2*, the methylation levels were 98.47% and 99.16% in the ON and OD (Figure 4E) groups, respectively.

## Discussion

Although the DNA methylation patterns in DMRs of imprinted genes in oocytes of offspring from mothers with pregestational diabetes [18] and/or obesity were not affected, DNA methylation modification was altered by maternal diabetes and/or obesity in spermatozoa of offspring. These results indicate that DNA methylation may play a role in passing the adverse effects from maternal diabetes and/or obesity on to the next generations.

Genomic imprinting involving DNA methylation is a widely existing epigenetic phenomenon in flowering plants and mammals [22-24]. Imprinted genes show parental-origin-dependent monoallelic expression controlled by differentially methylated regions (DMRs) [25-27]. The methylation at the DMRs which are erased in primordial germ cells (PGCs) and re-established during gametogenesis before being passed on to the next generation is germline-specific [28-30]. However, the time points of methylation establishment of germ cell imprints are different between males and females. In female germline, de novo methylation of imprints is initiated at the primordial germ cell (PGC) stage and mostly completed at the metaphase II stage (MII) after birth [31-33]. This process is earlier for males. *De novo* methylation in male PGCs of mammals takes place several days after erasure being completed at the stage of E14.5 to E16.5, and is mostly completed before the postnatal stage [34-36]. Therefore, the process of re-establishing imprints in the male germline is more easily disturbed by a deleterious uterus environment than that in the female germline. In the present study, we found the DNA methylation patterns at the DMRs of *H19* and *Peg3* in spermatozoa of offspring were influenced by pre-existing maternal diabetes and obesity, respectively. But they were not affected in oocytes of offspring born by mothers with diabetes and/or obesity [18].

It is well known that sexual dimorphism is very common phenomenon in mammals including humans. In humans, the expression of individual genes is different in the placenta between males and females [37,38]. A similar phenomenon is observed in the mouse [39,40]. McPherson and Chenoweth have discussed the evolution, benefits and costs of mammalian sexual dimorphisms [41]. We found that the effects on DNA methylation of imprinted genes were different in germ cells between female and male offspring born to mothers with diabetes and/or obesity. The sexual dimorphism may partly elucidate the different effects on DNA methylation of imprinted genes in germ cells of female and male offspring of diabetic and nondiabetic mothers. Certainly, the difference may also be induced by contamination with somatic cells. In our study, we showed that the difference was not the result of contamination with somatic cells: the methylation of *Peg3* (maternally imprinted) was altered in spermatozoa of offspring from obese mothers, but *H19* (paternally imprinted) was not changed. To further confirm this, we carried out the first-round PCR including primers of *Peg3* and s*H19*. Then they were amplified for the second-round PCR. We found that the alteration of *Peg3* was not induced by contamination with somatic cells.

In the present study, we found that the DNA methylation in DMR of *Peg3* was altered in spermatozoa of offspring from obese mothers but it was not affected in spermatozoa of offspring from diabetic mothers. However, the DNA methylation of *H19* in the offspring sperm was changed by maternal diabetes. This may be mainly because the two models have different influences on testis and sperm. It has previously been shown that maternal diabetes reduced testis weight, thickness of the testicular capsule, number of Leydig and Sertoli cells, number of spermatogonia, as well as sperm reserves and the sperm transit time through the epididymis in male offspring [42-44]. Although the offspring of obese mothers display sub-fertility, few reports are available on how maternal obesity affects testis function and sperm quality of offspring.

## Conclusions

In summary, the DNA methylation in spermatozoa of offspring born by obese and/or diabetic mothers was altered, indicating that abnormal DNA methylation modification in spermatozoa may play a role in the adverse effects passed on to the next generations by the mothers.

## Competing interests

The authors declare that they have no competing interest.

## Authors’ contributions

GZJ, ZCL, HS and SQY designed and wrote the manuscript. LQX, HY and GZJ acquired data. HZM analyzed the data. All authors read and approved the final manuscript.
